# Manually classified dataset of leaning and standing personnel images for construction site monitoring and neural network training

**DOI:** 10.1016/j.dib.2025.111516

**Published:** 2025-03-24

**Authors:** Alexandre Almeida Del Savio, Ana Luna Torres, Daniel Cárdenas-Salas, Mónica Vergara Olivera, Gianella Urday Ibarra

**Affiliations:** a Carrera de Ingeniería Civil, Instituto de Investigación Científica, Universidad de Lima, Lima, Peru; b Carleton University, Ottawa, Canada

**Keywords:** Construction site monitoring, Personnel image classification, Neural network training, Computer vision, Artificial intelligence, Safety monitoring in construction, Leaning and standing poses, Image dataset for AI, Construction workers, Pose classification

## Abstract

This data paper presents a manually labeled dataset of 1,214 images of personnel captured from a construction site using four static cameras. There are two classes, standing and people leaning. The classification is stored in accompanying text files and bounding box coordinates for every image. The compilation was done to support the developing and validation computer vision and AI models for construction site monitoring. This dataset addresses the challenges of finding personnel in different poses within complex construction environments. The resource will enhance construction site safety monitoring and personnel activity analysis by allowing more precise neural network training. The dataset is stored in a public repository, making it openly accessible for academic and industrial purposes regarding computer vision, civil engineering, and workplace safety.

Specifications Table

**Table:** 

Subject	Computer Science, Engineering
Specific subject area	Artificial Intelligence, Computer Science Applications, Computer Vision and Pattern Recognition, Civil Engineering
Type of data	Raw. Images .jpg Archives .txt
Data collection	The data were acquired from four static cameras around a construction site: one bullet-type camera, model IPC-HFW2831T-ZS-S2 [1], and three motorized IP PTZ cameras, model SD10A848WA-HNF [2]. The cameras were programmed using the DSSExpress-Base-License software [3]. The LabelImg v.1.8.1 tool [4] was used to classify images.
Data source location	All images were acquired from a building under construction at the Universidad de Lima, Lima, Peru. Lat. -12.084307°, Long. -76.971031°
Data accessibility	The data is hosted on a public and trusted repository. Repository name: Repositorio Institucional – Universidad de Lima Data identification number: Doesn't have Direct URL to data: https://doi.org/10.26439/ulima.datasets.19853 Instructions for accessing these data: The data is open access, anonymity is not compromised. The link to download the data set is presented in the section “Recurso(s) relacionado(s)”.
Related research article	Almeida Del Savio, A., Luna Torres, A., Cárdenas-Salas, D., Vergara Olivera, M.A., Urday Ibarra, G.T. (2023). Artificial Intelligence Applied to the Control and Monitoring of Construction Site Personnel. In: dell'Isola, F., Barchiesi, E., León Trujillo, F.J. (eds) Advances in Mechanics of Materials for Environmental and Civil Engineering. Advanced Structured Materials, vol 197. Springer, Cham. https://doi.org/10.1007/978-3-031-37101-1_2 [6]

## 1 Value of the Data

• Unique Dataset: This dataset consists of 1,214 high-resolution, manually classified images of construction personnel standing and leaning and addresses challenges in complex environments, such as variable lighting and occlusions. It can be challenging to obtain information from different activities at construction sites. These images can be of help to future researchers as a database of construction personnel and their activities to train their models or use along their databases.
• Improves Neural Network Training: Future research can use this data as a way to facilitate the development of accurate computer vision models for safety monitoring and activity analysis in construction sites.
• Interdisciplinary Applications: This is useful for research in civil engineering, AI, computer vision, and workplace safety, bridging technology and practical safety solutions.
• Educational and Industrial Relevance: Open access for academic learning and industrial innovation in safety compliance and ergonomic design. This information is accessible for other researchers for future projects regarding computer vision and pattern recognition.

## 2 Background

The classification of personnel within construction environments is a cardinal activity in the development of safety monitoring applications using computer vision. Del Savio et al. [7,8] targeted identifying “person” class within a construction site. However, most of these early methodologies were limited to precision, especially when individuals were captured running in diverse postures or different distances from cameras.

Building on these findings, Almeida Del Savio et al. [6] developed an improved methodology that categorized personnel into two classes: standing and leaning. The motivation for this is because construction sites are highly dynamic, with workers continuously changing poses depending on what they are doing. Given this subtle classification, the enhanced methodology greatly improved object detection and activity recognition precision.

This paper presents a dataset stemming from this extended methodology, tackling the challenges in the real construction environment. Issues such as cluttered backgrounds, changing light conditions, and high-resolution image analysis are resolved with care, guaranteeing the usability of this dataset in training neural networks. Integrating bounding box annotation also facilitates accurate personnel localization within the images for robust training and validation of computer vision models.

## 3 Data Description

**Fig. 1:**
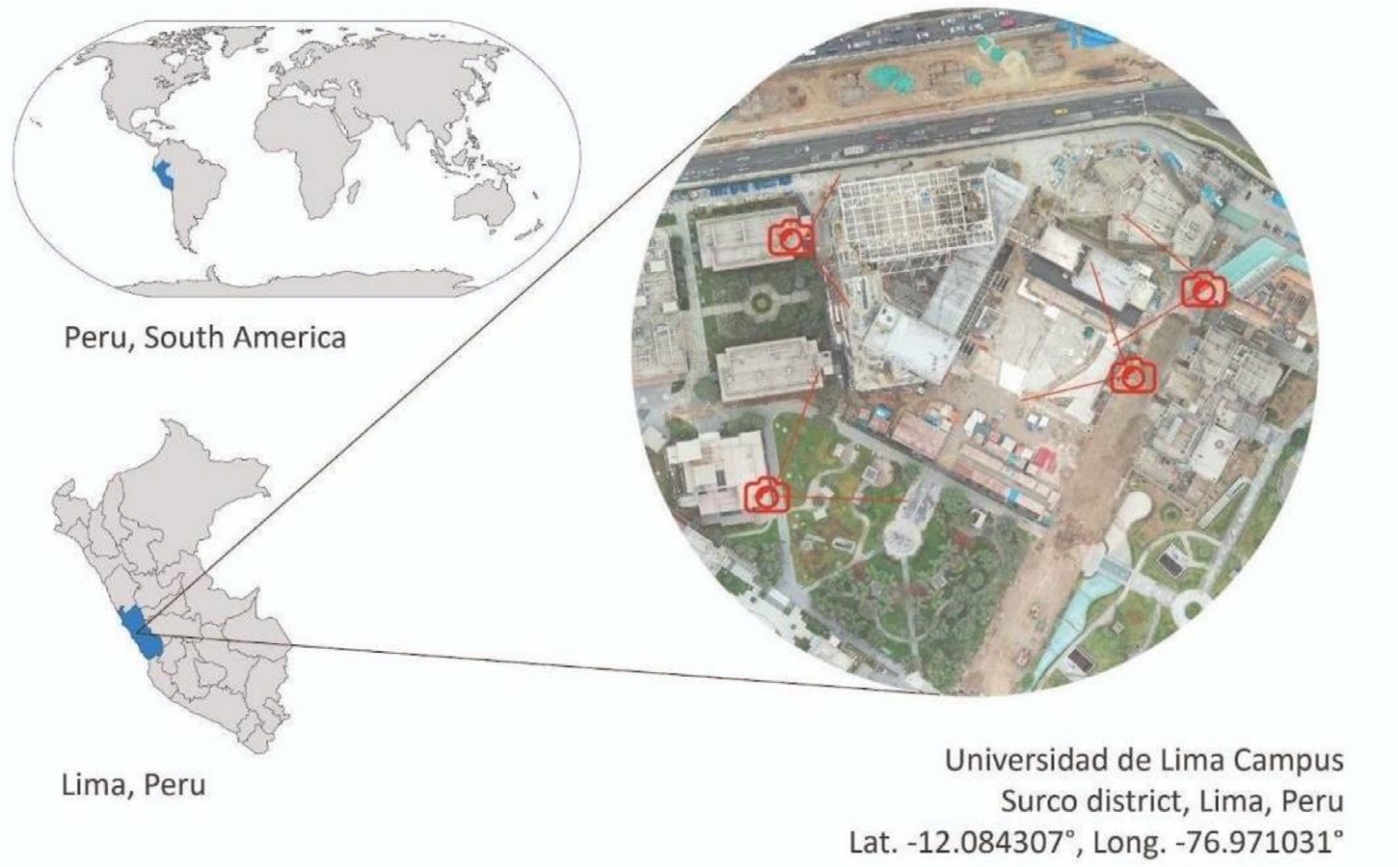
Construction site location [8].

The images were captured at the construction site of the Wellness Center building at the Universidad de Lima. 1,214 high-resolution images were extracted from footage recorded by four strategically placed static cameras (Fig. 1), ensuring comprehensive site coverage. This dataset was utilized by Almeida Del Savio et al. (2023) [6] to train artificial intelligence models for object detection in construction environments. The classification focused on two key categories of personnel: standing individuals (“person” [6,7]) and leaning individuals (“leaning_person”), as outlined in Table 1.

**Table 1:** Classes used for manual classification.

ID	Class	Object
0	“leaning_person”	
1	“person”	

Table 1 shows the classes used for manual classification and their respective images. These elements are found in the classes.txt file.

In the first column of Table 2, the original image displayed is the output of a frame extraction algorithm presented in [5], applied to video footage from the surveillance cameras. These images are in .jpg format with a 3840 × 2160 pixels resolution. The second column illustrates the manual classification process using the LabelImg v.1.8.1 software [4]. Objects in the images were annotated and categorized into their respective classes, with bounding boxes marked in green quadrilaterals. The results are in the third column, represented as .txt files containing structured metadata. This metadata includes the class ID (refer to Table 1) in the first column, the X and Y coordinates of the bounding box's top-left corner in the second and third columns, and the width and height of the bounding box in the fourth and fifth columns, respectively.

**Table 2:** Examples of selected images before and after manual classification, including the resulting .txt archive.

**Fig. 2:**
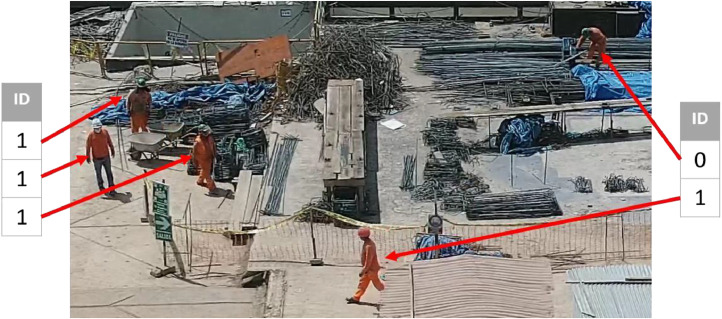
Extract of IMG-52.jpg's objects with their respective IDs.

Fig. 2 shows an extracted area from IMG-52.jpg (Table 2), where the objects are linked to their respective IDs according to Table 1.

## 4 Experimental Design, Materials and Methods

### 4.1 Experimental design

The research aims to develop a high-resolution image dataset for construction site monitoring and neural network training. The methodological design incorporates the following elements:

• Approach: A quantitative approach was adopted, leveraging numerical data and systematic classification methods to create a structured dataset [9].
• Purpose: This study fulfills a descriptive purpose [9] to document and classify personnel activities (standing and leaning poses) within a dynamic construction site environment. This serves as foundational data for neural network training.
• Design: A non-experimental design [9] was implemented. Images were collected passively without manipulating variables, ensuring authenticity in capturing real-world construction activities.

### 4.2 Materials

Cameras and Software:

Hardware: Four static cameras were employed:

• One bullet-type camera, model IPC-HFW2831T-ZS-S2 [1].
• Three motorized IP PTZ cameras, model SD10A848WA-HNF [2].

Software:

• Video footage was managed using DSS Express software [3].
• Frame extraction algorithm [5].
• Images were manually annotated with LabelImg v.1.8.1 software [4].

Data Specifications:

• Image format: .jpg
• Resolution: 3840 × 2160 pixels
• Metadata: Bounding box annotations stored in .txt files.

### 4.3 Methods

1. Data Collection

Images were captured from November 2020 to March 2021 at the construction site of the Wellness Center building at the Universidad de Lima (Lat. -12.084307°, Long. -76.971031°). Cameras were strategically positioned to ensure comprehensive coverage. Recording schedules avoided midday to minimize glare and ensure image clarity.

• Video Footage: Footage from each camera was recorded in 10-minute segments, with a frame extraction interval of 200 frames. Table 3 summarizes the video durations and extracted frames.

**Table 3:** Video footage duration and number of extracted frames per segment.

Video footage ID	Duration (mm: ss)	Extracted frames
VIDEO1.mp4	10:00	45
VIDEO2.mp4	10:00	45
VIDEO3.mp4	10:00	91
VIDEO4.mp4	10:00	90
VIDEO5.mp4	10:00	90
VIDEO6.mp4	10:00	90
VIDEO7.mp4	10:00	89
VIDEO8.mp4	10:00	90
VIDEO9.mp4	10:00	44
VIDEO10.mp4	10:00	90
VIDEO11.mp4	10:00	90
VIDEO12.mp4	10:00	90
VIDEO13.mp4	10:00	90
VIDEO14.mp4	10:00	90
VIDEO15.mp4	10:00	45
VIDEO16.mp4	10:00	45

2. Manual Annotation

Using the LabelImg v.1.8.1 software [4], personnel were classified into two categories:

• Standing Person (“person”) [6,7].
• Leaning Person (“leaning_person”) [6].

Each object was annotated with a bounding box, and the metadata was saved in corresponding .txt files, which include:

• Class ID
• Bounding box coordinates (X and Y for the upper-left corner, width, and height).

3. Environmental Conditions

Table 4 shows the date and time according to the ID of the image. Data collection accounted for varying lighting and temperature conditions, as shown in Table 5. Illuminance levels ranged from 37,000 lx to 90,000 lx, and temperatures ranged from 18°C to 26°C.

4. Validation

**Table 4:** Date and time allocation of image frames.

Image ID	Date (MM, DD, YYYY)	Time (24 hrs.)
IMG-1 – IMG-45	November 30, 2020	11:10 – 11:19
IMG-46 – IMG-135	November 18, 2020	13:29 – 13:39
IMG-136 – IMG-225	January 06, 2021	11:39 – 11:49
IMG-226 – IMG-315	January 06, 2021	11:39 – 11:49
IMG-316 – IMG-405	December 07, 2020	11:09 – 11:19
IMG-406 – IMG-495	November 20, 2020	11:29 – 11:39
IMG-496 – IMG-540	January 04, 2021	15:30 – 15:39
IMG-541 – IMG-585	January 06, 2021	15:39 – 15:49
IMG-586 – IMG-630	November 18, 2020	13:29 – 13:39
IMG-631 – IMG-721	December 07, 2020	11:09 – 11:20
IMG-722 – IMG-811	December 15, 2020	10:10 – 10:20
IMG-812 – IMG-901	January 04, 2021	15:29 – 15:39
IMG-902 – IMG-991	March 18, 2021	11:30 – 11:39
IMG-992 – IMG-1080	January 20, 2021	10:59 – 11:09
IMG-1081 – IMG-1170	March 08, 2021	11:30 – 11:39
IMG-1170 – IMG-1214	November 30, 2020	11:20 – 11:29

**Table 5:** Environmental conditions during image collection (Illuminance and temperature).

Date (MM, DD, YYYY)	Time (24 hrs.)	Illuminance (lx)	Air temperature (°C)
November 18, 2020	13:29 – 13:39	60000	18
November 20, 2020	11:29 – 11:39	62000	24
November 30, 2020	11:10 – 11:30	77700	21
December 07, 2020	11:09 – 11:20	37000	21
December 15, 2020	10:10 – 10:20	89000	23
January 04, 2021	15:29 – 15:39	61200	22
January 06, 2021	11:39 – 11:49	78000	24
March 08, 2021	11:30 – 11:39	90000	25
March 18, 2021	11:30 – 11:39	85000	26

The dataset was validated for completeness and consistency. Images were reviewed to ensure proper annotation and alignment with the defined classes.

## Limitations

This study acknowledges the following limitations that may influence the applicability and generalizability of the dataset:

• Fixed Camera Positions: All data was captured using fixed cameras only. Thus, the dataset is restricted to dynamic or mobile viewpoint scenarios. Further work on datasets may include mobile or drone-based captures for added versatility.
• Restricted Classification Categories: The dataset covers two critical personnel classifications, “standing” and “leaning.” While adequate for this study's objectives, it does not consider other broad classes of posture or related activities, such as walking, sitting, or operating equipment.
• Environmental Conditions: Data was collected at one construction site under specific environmental conditions, such as temperature and illuminance. These conditions may not represent diverse geographic or seasonal variations.
• Manual Annotation: While the annotations were rigorously performed, manual processes are prone to human error. Automating such tasks in the future could reduce potential inconsistencies and greatly improve scalability.
• Scope of Dataset: The dataset represents conditions and activities in a single construction site. If there are several sites, the variation in layout, equipment, and worker behavior will provide greater representation and applicability for this dataset.

These limitations provide opportunities for future work to build upon this dataset, expanding its applicability and addressing the challenges identified.

## Ethics Statement

This research did not involve human subjects, animal experimentation or social media platforms.

## CRediT authorship contribution statement

**Alexandre Almeida Del Savio:** Conceptualization, Validation, Resources, Writing – review & editing, Supervision, Project administration, Funding acquisition. **Ana Luna Torres:** Validation, Formal analysis, Visualization, Resources. **Daniel Cárdenas-Salas:** Methodology, Software, Validation, Formal analysis, Investigation. **Mónica Vergara Olivera:** Investigation, Data curation, Writing – original draft, Visualization. **Gianella Urday Ibarra:** Software, Investigation, Data curation, Writing – original draft.

## Data Availability

(Repositorio Institucional – Universidad de Lima)Dataset of manually classified personnel in images obtained from a construction site [Dataset] (Original data).

## References

[1] Dahua Technology, 8MP Lite IR Vari-focal Bullet Network Camera. (2024) https://www.dahuasecurity.com/Products/All-Products/Network-Cameras.

[2] Dahua Technology, 4K 48x Starlight+ IR WizMind Network PTZ Camera. (2024) https://www.dahuasecurity.com/Products/All-Products/PTZ-Cameras.

[3] Dahua Technology, DSS express. (2024) https://software.dahuasecurity.com/es.

[4] LabelImg v.1.8.1. (2024) https://pypi.org/project/labelImg/1.8.1/.

[5] Del Savio A.A., Luna Torres A., Cárdenas Salas D., Vergara Olivera M.A., Urday Ibarra G.T. (2023). Detection and evaluation of construction cracks through image analysis using computer vision. Appl. Sci..

[6] Almeida Del Savio A., Luna Torres A., Cárdenas-Salas D., Vergara Olivera M.A., Urday Ibarra G.T., dell'Isola F., Barchiesi E., León Trujillo F.J. (2023). Advances in Mechanics of Materials for Environmental and Civil Engineering. Advanced Structured Materials.

[7] Del Savio A.A., Luna Torres A., Cárdenas-Salas D., Vergara Olivera M.A., Urday Ibarra G.T. (2021). Proceedings of the International Conference on Artificial Intelligence and Energy System (ICAIES) in Virtual Mode.

[8] Del Savio A., Luna A., Cárdenas-Salas D., Vergara M., Urday G. (2022). Dataset of manually classified images obtained from a construction site. Data Brief.

[9] Del Savio A.A., Galantini Velarde K., Cáceres Montero L., Vergara Olivera M.A. (2024). Research training: unraveling the research methodological design challenge in engineering programs. Int. J. Eng. Pedagog. (iJEP).

